# Development of a Novel Compound Effective Against Juvenile, Adult, and Drug-Resistant *Schistosoma* Species

**DOI:** 10.3390/pharmaceutics17101268

**Published:** 2025-09-27

**Authors:** Sevan N. Alwan, Alexander B. Taylor, Stanton F. McHardy, Michael D. Cameron, Philip T. LoVerde

**Affiliations:** 1 Department of Biochemistry and Structural Biology, University of Texas Health at San Antonio, San Antonio, TX 78229, USA; taylorab@uthscsa.edu (A.B.T.); loverde@uthscsa.edu (P.T.L.); 2 Greehey Children’s Cancer Research Institute, University of Texas Health at San Antonio, San Antonio, TX 78229, USA; 3 Center for Innovative Drug Discovery, Department of Chemistry, University of Texas at San Antonio, San Antonio, TX 78249, USA; stanton.mchardy@utsa.edu; 4 Department of Molecular Medicine, UF Scripps Biomedical Research, Jupiter, FL 33458, USA; michaelcameron@ufl.edu

**Keywords:** oxamniquine, *Schistosoma*, drug discovery, drug resistance

## Abstract

Schistosomiasis, a neglected tropical disease affecting over 250 million people worldwide, relies on praziquantel (PZQ) as its sole treatment. However, PZQ has significant limitations, including inactivity against juvenile worms, inability to prevent reinfection, and emerging drug resistance. In this review, we outline the development of CIDD-0150303, a novel oxamniquine (OXA) derivative with pan-species and pan-stage activity against *Schistosoma mansoni*, PZQ-resistant *S. mansoni*, and *S. haematobium*. Using a structure-guided design approach, over 350 OXA analogs were synthesized and screened to identify leading drug candidate CIDD-0150303. CIDD-0150303 demonstrates 100% lethality in vitro and up to 80% reduction in worm burden in vivo. CIDD-0150303 is effective against both juvenile and adult parasites as well as PZQ-resistant *S. mansoni*. This compound represents a promising advance in schistosomiasis treatment to address urgent gaps in control/elimination strategies and PZQ resistance. However, dedicated safety and toxicity studies are still ongoing, and additional in vivo validation is required.

## 1. Introduction

Schistosomiasis is the second most prevalent parasitic infection in humans after malaria and remains a major neglected tropical disease. It affects over 250 million people in 78 countries, with up to 200,000 deaths annually and more than 800 million people at risk of infection [1,2,3]. Over 90% of the global disease burden occurs in sub-Saharan Africa, where *Schistosoma mansoni* causes intestinal–hepatic disease and *S. haematobium* causes urogenital disease [1]. *S. mansoni* causes chronic hepatic or intestinal illnesses and malnutrition in both Africa and Brazil, and it is suspected to be directly and indirectly involved in hepatocarcinogenesis [4,5,6]. *S. haematobium* is associated with urogenital conditions, including hematuria, bladder damage, and hydronephrosis. These conditions carry a risk of progression to severe diseases, such as kidney failure, bladder cancer, and infertility in the case of female genital schistosomiasis [7,8,9,10]. Systemic morbidities, including anemia, stunted growth, undernutrition, and decreased physical fitness, are also associated with *Schistosoma* infection [11].

The epidemiology of schistosomiasis is marked by a focal geographic distribution and infects all ages, with higher prevalence in children. Acute infection, a feverish syndrome, is most common in travelers following a primary infection. Travelers and soldiers are potentially at increased risk for infection, and most travel-associated cases of schistosomiasis are acquired in sub-Saharan Africa, where 90% of infections occur. Chronic schistosomiasis mainly affects those residing in endemic areas [12,13]. Although disease transmission is more common in tropical and subtropical areas, active transmission of urogenital schistosomiasis is observed in Southern Europe, a region previously thought to be schistosomiasis-free [14]. *Schistosoma* has a complex life cycle that involves freshwater snails as intermediate hosts with humans, among other mammals, as a final host. *Schistosoma* undergoes asexual proliferation in the snail, while sexual reproduction takes place in the vertebrate host, where three *Schistosoma* life stages are found: eggs, juvenile worms, and adult worms [15,16]. Schistosomes live from 3 to 10 years in the human host when humans encounter water infested with infectious-stage cercariae, which penetrate intact human skin, shed their forked tails, and form the juvenile stage [16,17]. The juvenile stage migrates throughout the body’s tissues through blood circulation, then through the lungs and heart to the vasculature of the liver, where maturation and mating occur. From here, the parasite couples migrate to their preferred egg-laying sites [18,19]. This journey takes about 45 days for *S. mansoni* and 72 days for *S. haematobium* [20,21,22,23]. Female schistosomes do not mature physically or reproductively unless they pair with the male. Upon pairing with a male worm, the male sends a signal to initiate and maintain female reproductive development [24,25]. For *S. mansoni*, each worm pair produces around 300 eggs each day [26]. About half of eggs produced reach the gut lumen and are released with feces into the environment. The other half are swept up in the circulation and trapped in the tissues, leading to severe clinical symptoms [15,27]. Here, the deposited eggs evoke a strong inflammatory response and become surrounded by a barrage of immune cells, generating granulomas. Thus, the eggs are the primary drivers of schistosomiasis-related pathology [19,28]. Due to the large overlap of *S. mansoni*- and *S. haematobium*-endemic regions in Africa, many people are at risk of co-infection. The distributions and risk factors for co-infections show little difference to overall single *S. mansoni* or *S. haematobium* infections. However, mixed schistosome species infection is understudied for human morbidity outcomes across endemic settings [29,30,31].

### 1.1. Current Treatment for Schistosomiasis

Presently, there is no vaccine available for this disease [13,32] and praziquantel (PZQ) is the only available treatment for schistosomiasis. PZQ has been indicated in the list of essential medications of WHO due to its effectiveness against all medically important *Schistosoma* species, in addition to other species of trematodes and cestodes [33,34,35,36]. The mainstay of schistosome control programs is repeated mass drug administration (MDA) chemotherapy with PZQ, currently 250 million doses per annum, which is used to treat at-risk and infected populations of human hosts [37,38,39,40]. PZQ is usually administered orally at dosages between 40 and 60 mg/kg in a single dose or two doses depending on the level of infection [35]. In vitro, adult schistosome treatment with PZQ leads to muscular paralysis, vacuolation, and blebbing of the tegmental and sub-tegmental structures due to rapid influx of Ca^2+^ [41]. The precise mode of action was not completely understood until recently. It is now known that PZQ activates a flatworm transient receptor potential channel (TRPMPZQ) to mediate sustained Ca^2+^ influx and worm paralysis [42,43,44]. MDA with PZQ has brought about reductions in both urogenital and intestinal schistosomiasis. However, some locations have maintained high levels of infection prevalence and intensity due to the imperfect efficacy of the drug against adult schistosomes [45,46]. For example, persistent hot spots in Kenya have been described because drug administration strategies were less effective at reducing prevalence and intensity in those areas after 5 years of treatment compared to other villages [47]. In a 2022 study, despite 12 rounds of school-based preventive chemotherapy, the worm burden remained at 52.7% [48]. Another study reported 60–90% cure rates in sub-Saharan Africa, where 90% of infections occur [49]. This mono-chemotherapeutic strategy for schistosomiasis control presents challenges as the development of PZQ-resistant parasites remains a continuous threat, and evidence for drug resistance in the field and laboratory has been reported [50,51,52,53,54]. Moreover, PZQ does not prevent reinfection, and it is not active against juvenile-stage schistosomes. The failure to eliminate liver-stage worms results in their rapid progression to the adult egg production stage within 2 to 3 weeks after PZQ treatment, highlighting the urgent need for a more sustainable approach [51,55,56,57,58,59]. This treatment limitation perpetuates transmission in endemic areas where infected people are receiving annual MDA. The presence of juvenile schistosomes in humans effectively postpones the emergence of egg-patent infection, thereby impacting transmission dynamics [15,60]. A review by Zdesenko and Mutapi has shown additional factors related to metabolic and pharmacokinetic problems that can result in variable levels of the drug in systemic circulation, potentially contributing to low treatment cure rates [61]. Due to PZQ treatment limitations and constant selection pressure, there is an urgency for developing new therapies to reach WHO goals for the elimination of schistosomiasis as a public health problem. A recent modeling study simulating mass drug administration control programs provides guidance for novel drug development for schistosomiasis. The study revealed that novel anthelmintic drugs that can kill both adult and juvenile schistosomes with higher efficacy than praziquantel could provide public health gains in control programs for schistosomiasis, especially in high-burden settings [62]. About 200 PZQ derivatives have been developed since PZQ became available for general use. Most of them demonstrated low efficacy, while others, like antimony derivatives, were excessively toxic and abandoned [63]. Artemisinin-derivative drugs were shown to have efficacy against juvenile schistosomes, thereby promoting interest in further development because the drugs are also used against malaria. However, developing alternatives for widespread use in areas that are co-endemic for both schistosomiasis and malaria could lead to increased resistance in malaria parasites [64,65].

### 1.2. Historical Treatment for Schistosomiasis

Previous treatments for *S. mansoni* included oxamniquine (OXA) and hycanthone (HYC). OXA was used extensively [66,67] until the patent on PZQ expired, after which PZQ usage became more prevalent [38,68]. Similar to PZQ, which lacks efficacy against juvenile schistosomes [69,70], OXA is only effective against the adult worm stage of *S. mansoni.* HYC is effective against the adult worm stage of *S. mansoni* and *S. haematobium* but has been shown to be a carcinogen [71,72,73] and has fallen out of use. About 9 million schistosomiasis patients have been treated in South America, the Caribbean area, the Middle East, and Africa [66,67,74]. Pfizer developed OXA and made it available for general use in 1975 [74,75]. OXA treatment shows minor side effects and is well tolerated by patients, with a high cure rate when administered as a single oral dose. It usually utilizes a single oral dose strategy at 20–40 mg/kg [76]. Higher doses above 60 mg/kg in a series of two or three oral doses have also been used to obtain the desired therapeutic efficacy in regions including Egypt, South Africa, and Zimbabwe. The drug is well absorbed orally [74] and has a half-life of about 1.5–2 h [77]. Systemic OXA is metabolized by oxidative processes and excreted in the urine without presenting negative effects. OXA is not recommended for patients with epilepsy [66]. Unlike PZQ, which is active against the three main *Schistosoma* species, OXA fails to treat infections by *S. haematobium* or *S. japonicum*, and the female worms are less sensitive to OXA treatment than males. In addition, evidence of drug resistance against OXA in the laboratory and in the field has been reported [78,79]. Efforts to derivatize OXA through structural modifications aimed at producing more efficacious compounds were carried out mostly between 2002 and 2017 [63,80,81,82]. However, most of the derivatives still exhibited issues with toxicity, poor solubility, or limited activity [63]. Encouragingly, a series of studies [83,84,85] has demonstrated the efficacy of OXA derivatives against *S*. *mansoni* both in vitro (100% killing) and in vivo (100% killing with a 200 mg/kg dose) and *S*. *haematobium* in vitro (75% killing activity). Hess et al. (2017) synthesized ruthenocenyl-, ferrocenyl-, and benzyl-based organometallic OXA conjugates to improve ADME and physicochemical properties [83]. Current studies focus on improving bioavailability to improve in vivo activity [85]. OXA has a high production cost, partially due to a biotransformation hydroxylation process, and the supply is limited because it is no longer manufactured by Pfizer. Some of the OXA-inspired analogs now benefit from a simple and cost-effective synthesis strategy [86].

It was not until 2013 that Valentim et al. identified the precise mechanism of action of OXA [87]. This was an important step in developing new OXA derivatives using structure–activity relationships (SARs). OXA is a prodrug that is enzymatically activated within the parasite [88]. It binds to a specific *S. mansoni* sulfotransferase (SULT), where it is transiently sulfated, resulting in its activation [23]. SULTs are enzymes that catalyze the transfer of a sulfuryl group (SO_3_) from the cofactor 3′-phosphoadenosine 5′-phosphosulfate (PAPS) to substrates. Activated OXA binds to DNA and other macromolecules, resulting in the killing of the schistosome adult worms [88].

## 2. Rational Approach to Drug Discovery for Human Schistosomiasis

**Figure 1:**
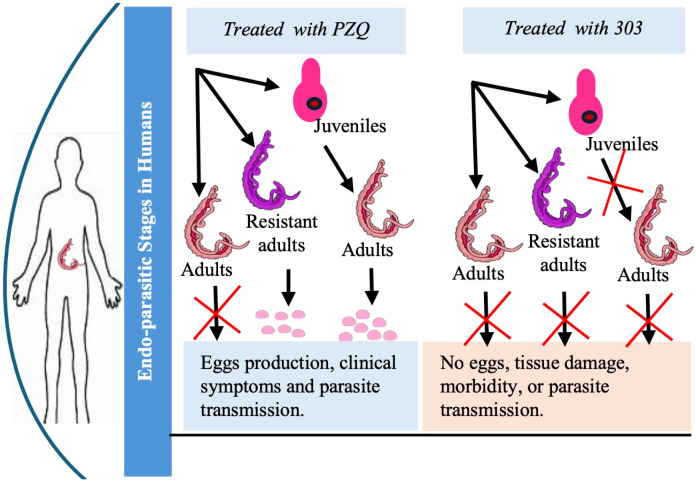
Comparative research schema showing the effects of PZQ versus compound CIDD-0150303 on juvenile, adult, and drug-resistant *Schistosoma* and the impact on egg production.

The goal of this review is to present a novel anthelmintic drug development that can kill adult, juvenile, and resistant schistosomes as a robust alternative to, or combination therapy with, PZQ to mitigate resistance development and improve efficacy in high-burden settings (Figure 1). A structure-guided approach was used to modify OXA in pursuit of a novel anthelmintic drug designed to surpass PZQ in its impact on disease control, thus contributing to greater public health gains in schistosomiasis control programs.

### 2.1. Oxamniquine as a Starting Point for Novel Anthelmintic Design

**Figure 2:**
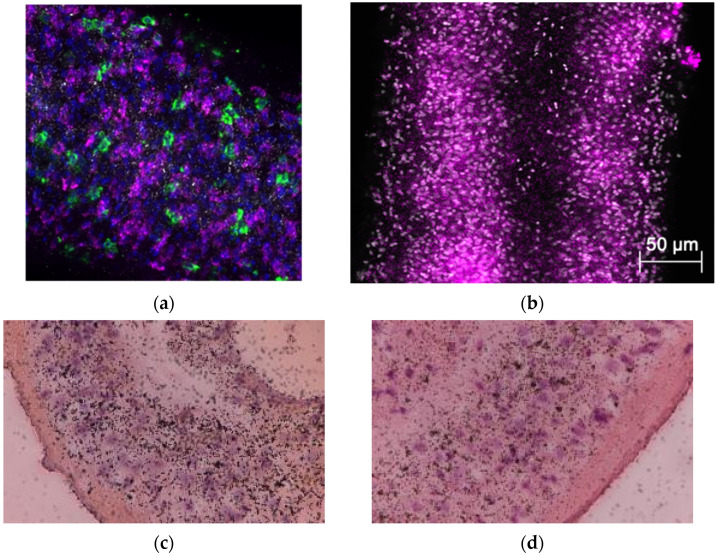
In situ localization of *S. mansoni* SULT and [3H] OXA localization. Freshly collected male worms of (**a**) *S. mansoni* and (**b**) *S. haematobium.* Body sections were fixed in 4% formaldehyde, dehydrated, and hybridized with riboprobe. A and B panels show that SULTs were produced by cells throughout the entire worm (blue is DAPI; green is stem cells (positive control); and magenta is *S. mansoni* SULT). [3H] OXA was incubated with adult male worms for 45 min, washed 3 times, and incubated for 14 days, with groups of 10 worms being removed and fixed in 4% paraformaldehyde every two days. Fixed worms were embedded in paraffin, sectioned, and subjected to autoradiography. Slides were lightly stained with H&E. The silver grains represent activated OXA bound to macromolecules either as an electrophile or neutrophile. The clustering is less prominent with longer time post-OXA exposure for (**c**) day 6 and (**d**) day 12 [89].

OXA was selected as the foundation for drug development due to its established anthelmintic activity, providing a valuable scaffold for designing more effective compounds with improved efficacy and broader impact in *Schistosoma* control. Despite being effective against only *S. mansoni* and the evidence for drug resistance [78,79], OXA is an attractive compound to develop because (1) OXA is not toxic to human cells, which lack enzymes to convert OXA into a toxic form; (2) OXA’s mode of action is different than PZQ, which will enable killing of PZQ-resistant parasites; and (3) OXA binds into the SULT binding pocket of the major two species, which is critical for our drug development approach. Whole-worm in situ hybridization on *S. mansoni* and *S. haematobium* was performed to determine the number and location of cells expressing the SULT. SULTs were expressed by cells throughout the entire worm. To identify where activated OXA binds within the schistosome after activation, male worms were treated with [3H] OXA (Figure 2). After a single 45 min exposure, as shown in the right panel, radiolabeled OXA was incorporated throughout the multicellular eukaryote even days after [3H] OXA exposure to the parasite, which subsequently led to parasite death [89,90,91]. Today, the precise mode of action for both drugs is well understood. A SULT ortholog is also expressed by *S. haematobium* [92], which raises the following question: Why is oxamniquine an effective drug against *S. mansoni* and not *S. haematobium*? As demonstrated by LoVerde et al. [93], sequence variation among the three SULT orthologs did not prohibit OXA binding in the active sites. Rather, it was found that OXA could be soaked into *S. haematobium* crystals and was observed bound in the active site. Further, SULT from *S. haematobium* has activity against OXA, although at lower catalytic efficiency compared to *S. mansoni*. Therefore, the answer to the question is that OXA does not fit into the SULT binding pocket from *S. haematobium* productively and does not get activated to a sufficiently toxic level [92,94].

### 2.2. Design and Synthesis of Modified OXA and Choice of Lead Compound

**Figure 3:**
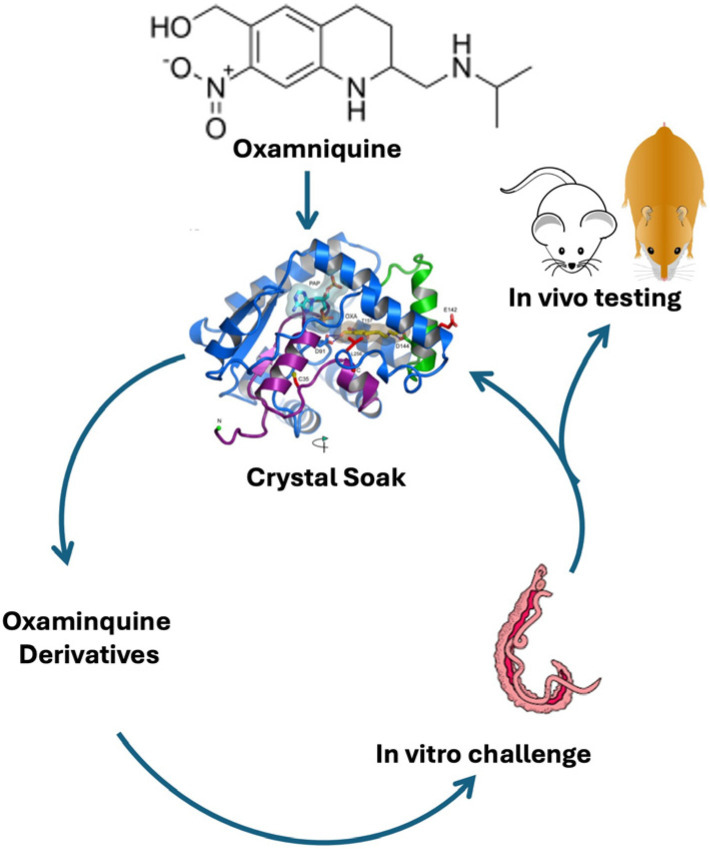
Iterative process of identifying new drugs [93].

Guided by data from X-ray crystallographic studies and *Schistosoma* worm-killing assays on OXA, the structure-based drug design approach produced a robust SAR program that identified several new lead compounds with effective worm-killing abilities [70,86,92,93,95]. OXA was soaked into SULT crystals, and the structural relationships were determined to guide the synthesis of derivatives. The OXA derivatives were tested for cidal activity using worm motility assays or direct observation in cultured parasites. Derivatives that showed the best cidal activity were soaked into new crystals, and the process was repeated (Figure 3) [93,95]. Structures for *S. mansoni* SULT (*Sm*SULT) and *S. haematobium* SULT (*Sh*SULT) were used to design highly efficacious OXA derivatives that will kill the two major species of *Schistosoma*.

**Figure 4:**
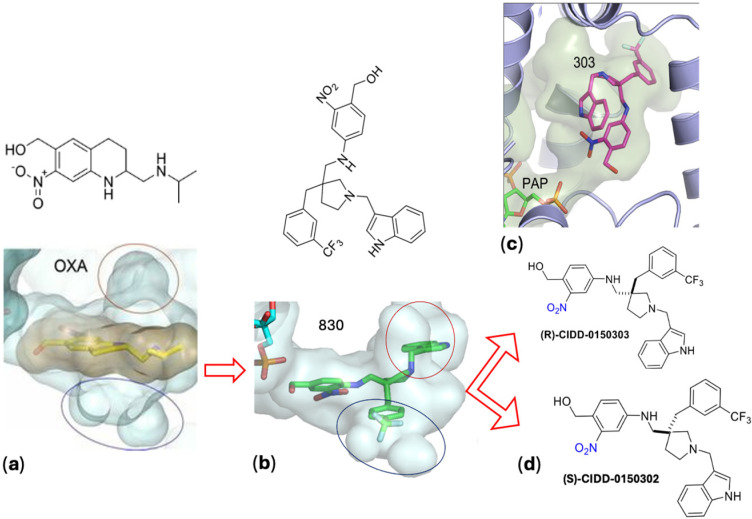
Evolution and identification of lead compounds. (**a**) The design strategy involved modifying OXA by removing the element of rigidity in the tetrahydroisoquinoline ring system, introducing two rotatable bonds, and adding a new substituted pyrrolidine ring to allow three-point SAR studies to optimize *Sm* SULT and *Sh* SULT residue interactions. The OH group in the upper left corner of the molecules is the site of sulfation, resulting in the formation of a reactive product that alkylates schistosome DNA and other macromolecules in sensitive parasites. This strategy resulted in the discovery of (**b**) CIDD-0149830, which was separated to provide (**c**) CIDD-0150303 and (**d**) CIDD-0150302. Images were generated using PyMOL (The PyMOL Molecular Graphics System, Version 3.0 Schrödinger, LLC, New York, NY, USA) [70,86,92].

More than 350 novel analogues of OXA were designed, synthesized, and screened (Figure 3). Several lead derivatives that show 100% pan-specific killing activity in vitro have been identified. The hypothesis was to access the two cavities identified in SULT crystal structures (Figure 4a) that were not occupied by OXA. Through multiple rounds of medicinal chemistry, the design strategy resulted in the discovery of CIDD-0149830 (Figure 4b), which demonstrates antischistosomal activity across three of the *Schistosoma* species [70,95]. To support these efforts, convergent and efficient syntheses to this chemical series were developed, allowing manipulation of multiple functional groups in parallel to synergize schistosomal SAR with optimization of “drug-like” physicochemical properties to access the two lipophilic cavities. Routine separation of the two enantiomers of CIDD-0149830, which was easily executed by preparative chiral HPLC methods, provided CIDD-0150303 (Figure 4c) and CIDD-0150302 (Figure 4d), of which the active enantiomer CIDD-0150303 was observed to have robust and pan-active worm-killing ability. CIDD-0150303 was identified as the compound with the most antischistosomal activity, with efficacy against the two major human schistosomes and against both mature and juvenile worms. It has been shown that CIDD-0150303 has a different mode of action than PZQ and was able to kill PZQ-resistant parasites in animal studies [70,93,95]. In selecting compounds to pursue, the most important test criteria were pan-species killing and lethal activity against both adult and juvenile parasite stages, which helped confirm the mechanism of drug action and dose response.

## 3. Proof of Principle

### 3.1. CIDD-0150303 Dual Efficacy

**Figure 5:**
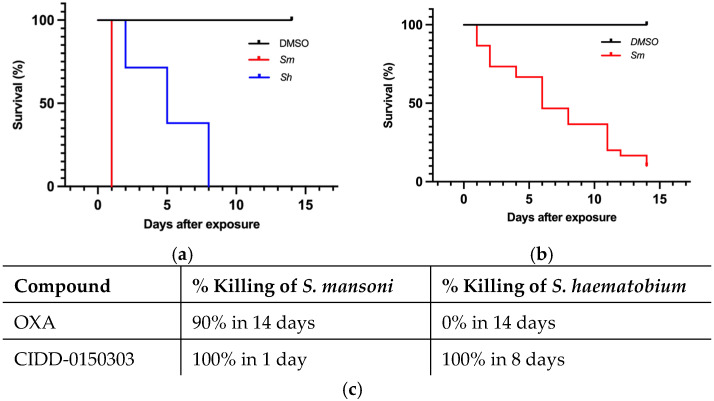
Kaplan–Meier curves demonstrating CIDD-0150303 and OXA lethality at 143 µM. CIDD-0150303 (**a**) and OXA (**b**) were tested against adult male worms in vitro (**c**) percentages of worm killing. Both compounds were administered at a final concentration of 143 µM per well for 45 min and subsequently washed 3X with fresh medium. All assays were performed in 3 experimental and biological replicates. Survival was plotted as a percentage over time using Kaplan–Meier curves. Pairwise comparison was performed using a log-rank test with Bonferroni correction for multiple testing. The *p*-value for OXA and CIDD-0150303 compared to DMSO was <0.001 [70].

**Figure 6:**
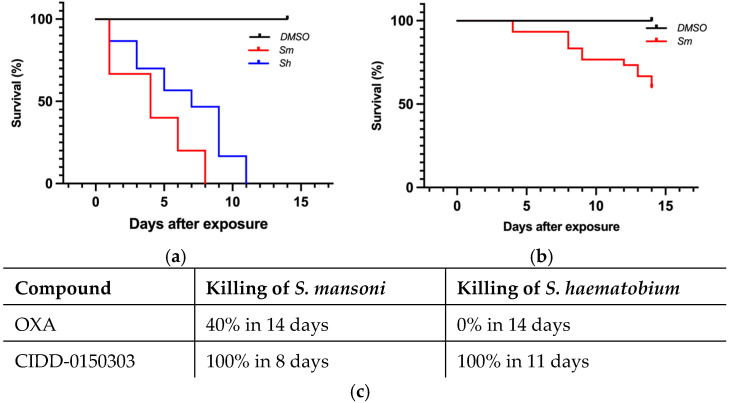
Kaplan–Meier curves demonstrating CIDD-0150303 and OXA lethality at 71.5 µM. CIDD-0150303 (**a**) and OXA (**b**) were tested against adult male worms in vitro (**c**) percentages of worm killing. Experiments and analyses were performed as described in Figure 5 [70].

**Figure 7:**
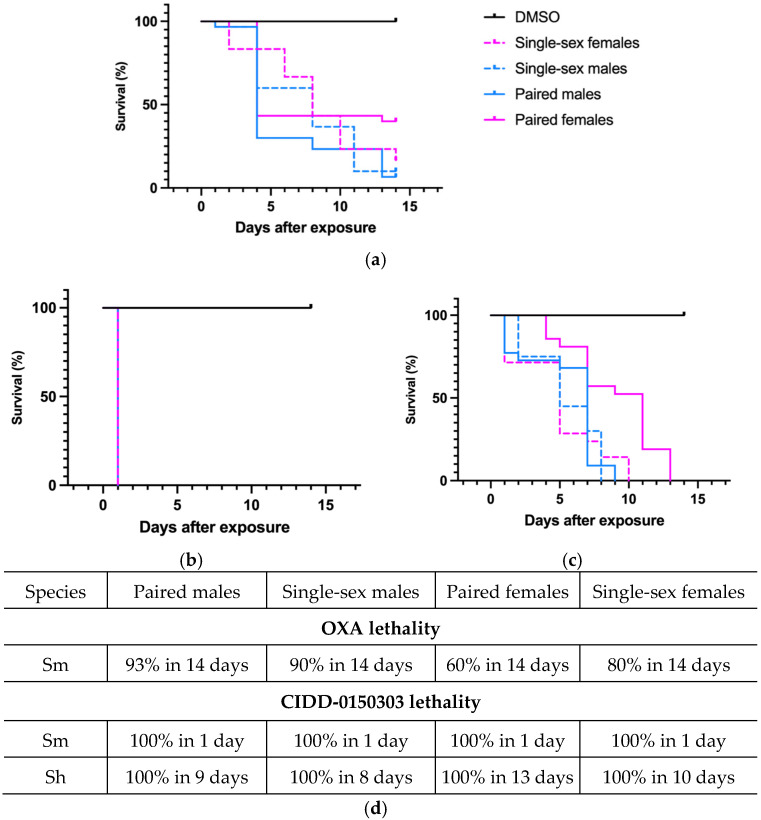
Kaplan–Meier curves demonstrating the ability of OXA and CIDD-0150303 to kill both genders. (**a**) OXA against both genders of *S. mansoni*. (**b**) CIDD-0150303 against both genders of *S. mansoni*. (**c**) CIDD-0150303 against both genders of *S. haematobium*. (**d**) percentages of worm killing. OXA and CIDD-0150303 were tested against 10 adults of a single sex—female and male worms and female and male worms in worm pairs per well. Both compounds were solubilized in 100% DMSO and administered at a final concentration of 143 μM per well for 45 min and washed 3X with media. The 45 min exposure mimics the exposure time in a human. All screens were performed in experimental and biological triplicates. Survival was plotted as a percentage over time using the prism/curve comparison/log-rank (Mantel–Cox) test. The *p*-value threshold for each derivative compared to DMSO was <0.00 [70].

CIDD-0150303 demonstrated 100% pan-specific killing activity in vitro. For comparison, OXA and CIDD-0150303 were tested against adult schistosomes at concentrations of 143 µM or 71.5 µM [70]. CIDD-0150303 was tested in three replicates (10 worms per well) and evaluated with DMSO (no drug control) and OXA (parent drug). The compounds were incubated with adult worms for 45 min, mimicking physiological conditions in humans [86,93,95]. CIDD-0150303 was the best derivative, as it killed 100% of *S. mansoni* and *S. haematobium* within 7 days at 143 µM compared to OXA, which only killed 90% of *S. mansoni* in 14 days (Figure 5). CIDD-0150303 also killed 100% of *S. mansoni* and *S. haematobium* at 71.5 µM compared to OXA, which only killed 40% of *S. mansoni*. Each condition contained 10 worms per well, performed in three independent biological replicates. The results and the 95% confidence intervals are shown in Figure 6. In addition, CIDD-0150303 exhibited potent lethality against both male and female *Schistosoma* parasites in similar conditions. At a concentration of 143 μM, CIDD-0150303 killed 100% of unpaired female and male worms from bi-sex infection, as well as female and male worms in worm pairs. Interestingly, CIDD-0150303 killed both genders within 24 h (Figure 7) in contrast to OXA, which showed greater efficacy against male worms. The sex-based difference in OXA susceptibility is associated with higher expression levels of *Sm*SULT in adult male worms compared to females [70,89]. These findings suggest that the use of CIDD-0150303 may allow for a lower optimal dose while still achieving high levels of parasite killing. Notably, CIDD-0150303 was able to induce 100% mortality of both single and paired adult male and female worms from both species within a short time frame.

**Figure 8:**
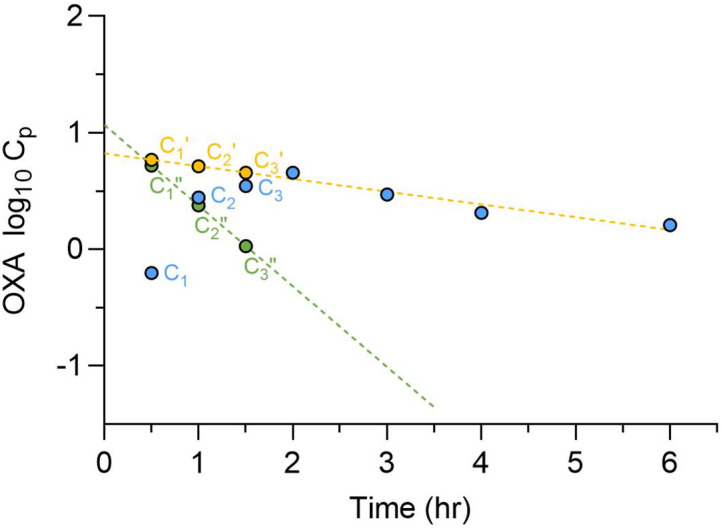
Determination of OXA oral absorption rate constants and portal concentration. Observed OXA plasma concentration-time profiles after a single 1000 mg oral dose in Sudanese patients (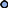, C) were plotted on a semi-logarithmic scale, and the terminal phase was extrapolated to the *Y*-axis. Patient plasma concentration values (C) were subtracted from the corresponding concentration from the extrapolated terminal line (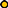, C’, **---**). The residual line was constructed by plotting the difference (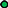, C”, **---**) between the extrapolated and the observed concentrations for each timepoint in the absorption phase. The absorption rate constant was determined from the slope of the residual line. The calculated total hepatic inlet concentrations of OXA in patients was 94 µM [98].

C_max_ concentrations from clinical dosing typically range between 4 and 15 µM [74,77,96,97]; however, in vitro studies have consistently shown limited efficacy at these levels. Notably, *Schistosoma* worms residing in the portal vasculature are exposed to much higher drug concentrations during the absorption phase. A study conducted in Sudan reported plasma OXA concentrations following a 1000 mg oral dose in both patients and healthy controls [75]. This dataset was reanalyzed [98] to estimate OXA concentrations in the portal vein, determining a value of 94 µM after a 1000 mg dose, representing the lower end of the clinical dosing range (Figure 8). Similarly, a 100 mg/kg oral dose in mice yielded a portal concentration of 143 µM, which models the exposure from a 30 mg/kg dose in humans. In in vitro worm-killing assays, worms were exposed to OXA at concentrations and durations (45 min) that mimic portal vein levels corresponding to a 30 mg/kg human dose. This serves as a baseline for optimizing more potent worm-killing compounds.

**Figure 9:**
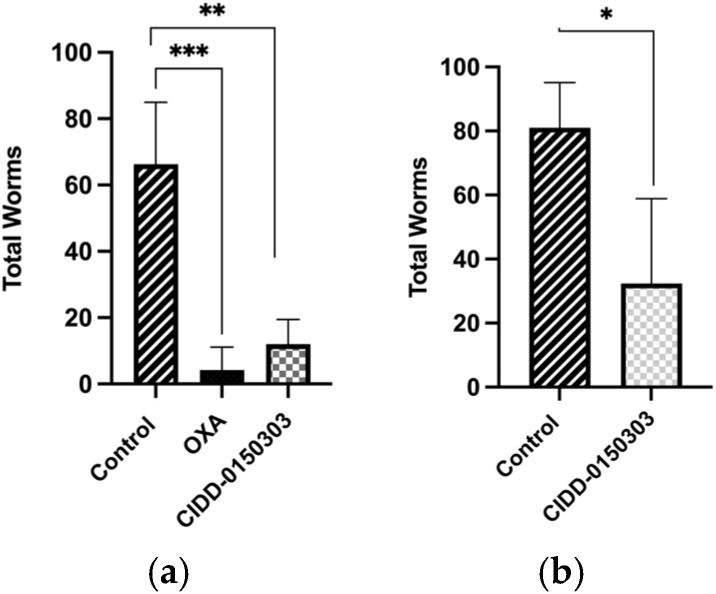
Effect of CIDD-0150303 on animals infected with (**a**) *S. mansoni* and (**b**) *S. haematobium*. Five mice per group were infected with *S. mansoni***,** and five hamsters per group were infected with *S. haematobium*. Worms were collected 14 days after treatment with a single dose of 100 mg/kg by oral gavage and compared to the untreated control group. CIDD-0150303 reduced the worm numbers of *S. mansoni* to 81.9% (*p* = 0.0061) and *S. haematobium* to 71.2% (*p* = 0.0672) compared to the negative control (95% CI). Prism/unpaired *t*-test (*p* < 0.05). *p* < 0.05 (*), *p* < 0.01 (**), *p* < 0.001 (***) [70].

Follow-up animal experiments were performed to evaluate the efficacy of CIDD-0150303 against *S. mansoni* and *S. haematobium*. Five BALB/C mice ((Envigo, Indianapolis, IN, USA) were infected with 80 *S. mansoni* larvae (cercariae) and treated with 100 mg/kg of drug by oral gavage at 45 days post-infection (dpi). Worms were perfused, harvested, and counted at 59 dpi to determine the worm burden reduction (Figure 9a). To test CIDD-0150303 against *S. haematobium*, hamsters (Envigo, Indianapolis, IN, USA) were infected with 100 larvae and treated 90 days later with 100 mg/kg of CIDD-0150303 orally (Figure 9b). CIDD-0150303 reduced the worm numbers of *S. mansoni* to 81.9% (*p* = 0.0061) and *S. haematobium* to 71.2% (*p* = 0.0672) compared to the negative control (95% CI). However, OXA demonstrated higher lethality in animals, prompting an optimization plan to increase in vivo worm killing through increased oral bioavailability and portal drug concentration of CIDD-0150303 [70]. A summary of in vivo worm burden reductions is provided in supplementary Table S1.

Published studies demonstrate that derivatives have been produced that kill 100% of *S. mansoni* and *S. haematobium* in vitro and reduce worm burden by up to 80% in animal models [70,93,95]. A dual efficacy against *S. mansoni* and *S. haematobium* would be most beneficial, since they are prevalent in different parts of the world. In sub-Saharan Africa, where 90% of *Schistosoma* cases occur, *S. mansoni* and *S. haematobium* are both endemic, and co-infection is common [30]. Therefore, a single drug that is efficacious against both is consequential.

**Figure 10:**
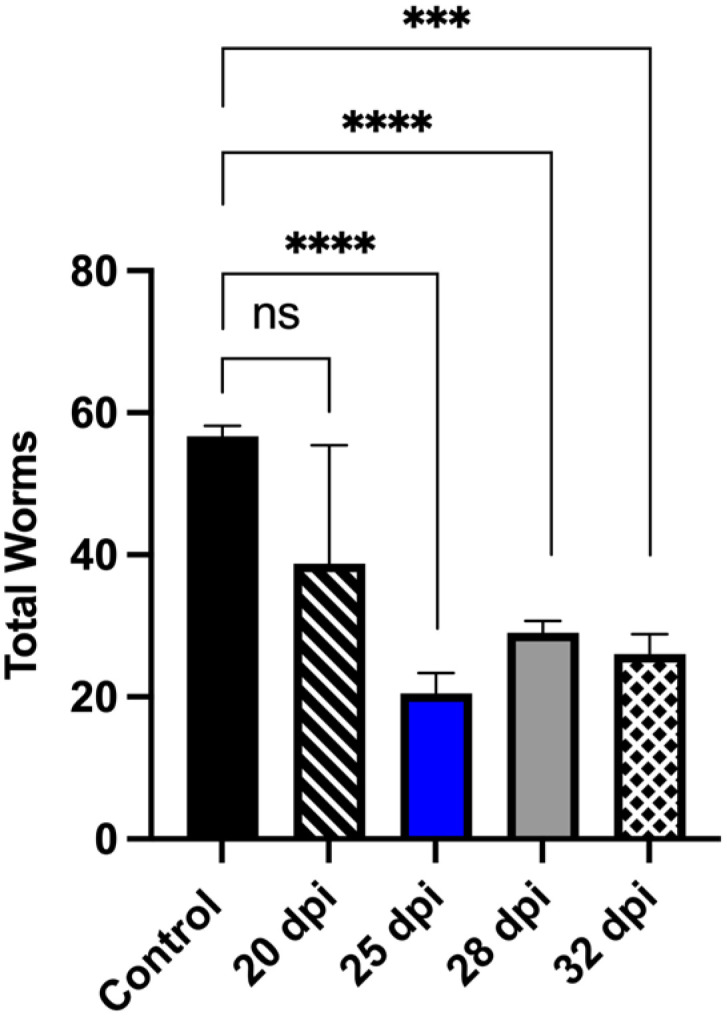
CIDD-0150303 significantly reduced the number of collected *S. mansoni* juvenile worms from infected mice. Five mice per group were infected with 80 cercaria of *S. mansoni*. Mice were treated with a single dose of 100 mg/kg by oral gavage of CIDD-0150303 on the day specified on the *X*-axis, and worm burden was determined at 45 dpi. Prism/unpaired t-test (*p* < 0.05). CIDD-0150303 reduced the worm burden significantly on 25 dpi by 64.8% (*p* = 0.0001), 28 dpi by 48.9% (*p* = 0.000), and 32 dpi by 54.1% (*p* = 0.0005) (95% CI). Prism/unpaired *t*-test (*p* < 0.05). *p* < 0.001 (***), *p* < 0.0001 (****). ns, not significant [70].

In heavily endemic areas, where 90% of schistosomiasis cases are caused by *S. mansoni* and *S. haematobium*, the adult worms of both species are killed by PZQ. However, liver-stage worms are unaffected and will continue to develop into egg-laying adults within weeks of re-establishing the infection and maintaining egg production and tissue pathology [99]. To add value to chemotype discovery efforts, derivatives that kill immature, liver-stage schistosomes have been the focus of further research. CIDD-0150303 killed 100% of juvenile worms from *S. mansoni* and *S. haematobium* in an in vitro assay. These results were tested in an animal model to evaluate the efficacy of CIDD-0150303 against juvenile liver stage parasites. CIDD-0150303 was effective against juvenile worms at 25 dpi when five *S. mansoni-*infected mice were treated with 100 mg/kg orally, and the worm burden reduction was 64.7% (*p* = 0.0001) with 95% CI (Figure 10) [70]. Drugs that can kill liver-stage worms are a significant advance over PZQ. A summary of in vivo worm burden reductions is provided in supplementary Table S2.

### 3.2. Evidence That the Mode of Action of PZQ Differs from CIDD-0150303

**Figure 11:**
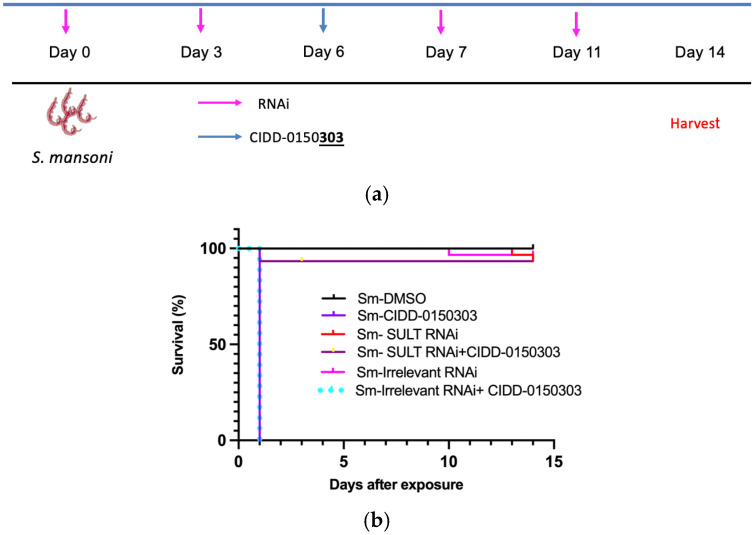
Kaplan–Meier curves demonstrating the (**a**) experiment schedule (**b**) knockdown of *S. mansoni* SULT confers resistance upon challenge. Adult male schistosome parasites, 10 per well, were treated with either *Sm* SULT dsRNA or dsRNA of an irrelevant gene at a final concentration of 30 µg/mL on days 0, 3, 7, and 11. The worms were treated with CIDD-0150303 for 45 min on day 6. The worms were observed for 14 days, pooled, and quick-frozen. The treatment groups were as follows: *Sm* SULT dsRNA alone, *Sm* SULT + CIDD-0150303, irrelevant gene control dsRNA, CIDD-0150303 alone, and DMSO solvent alone. All experiments were performed in 3 replicates. After 14 days, worm killing was observed in the presence of CIDD-0150303 with or without irrelevant gene control dsRNA. *Sm* SULT RNAi alone, irrelevant RNAi, and *Sm* SULT RNAi + CIDD-0150303 had 93%+ survival and displayed healthy characteristics. All other groups expressed similar, expected sensitivity levels to CIDD-0150303 treatments [70,89].

The mechanism of action of CIDD-0150303 is through enzymatic sulfation. RNA interference (RNAi) against *S. mansoni* SULT was used to test the ability of CIDD-0150303 to kill schistosomes [70]. Worms with knocked-down SULT expression were resistant to CIDD-0150303-induced killing (Figure 11). Each condition contained 10 worms per well and was performed in three biological replicates, with 95% confidence intervals shown. Quantitative polymerase chain reaction (qPCR)-verified RNAi treatment resulted in mRNA knockdown of approximately 99%. This demonstrates that the previously identified SULT is required for activating CIDD-0150303 [89].

### 3.3. Determining Whether CIDD-0150303 Demonstrates Antischistosomal Efficacy in Resistance Settings

**Figure 12:**
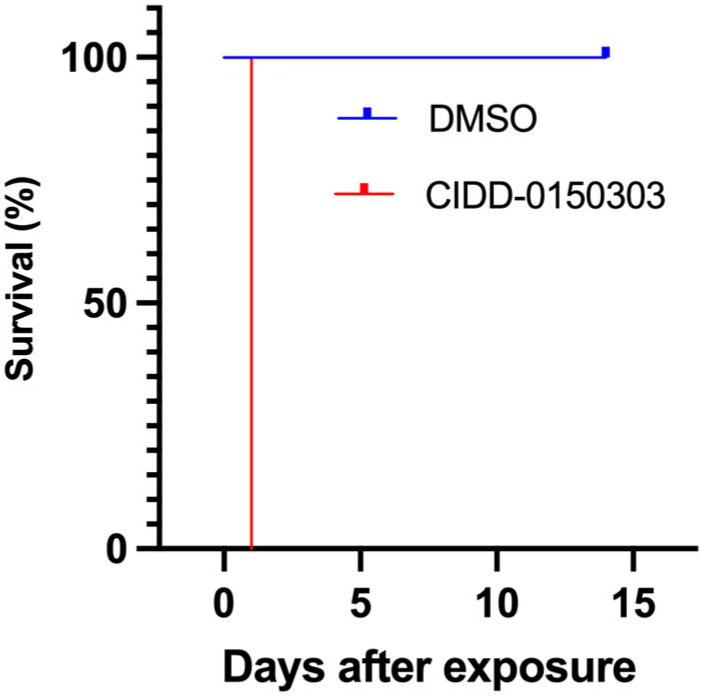
Kaplan–Meier curves demonstrating the ability of CIDD-0150303 to kill *S. mansoni* PZQ-R. Experiments and analyses were performed as described in Figure 5 [70].

PZQ resistance is the most compelling reason for the development of an additional treatment, as OXA resistance alleles have been identified from field studies in some areas of Brazil [100]. Previously, it was demonstrated that the mechanism of action of CIDD-0150303 is similar to OXA, confirming that the mode of action is conserved [87]. A PZQ-resistant (PZQ-R) schistosome strain is available, and published data demonstrated that CIDD-0150303 killed 100% of PZQ-R parasites in vitro (Figure 12). The IC_50_ for the PZQ-R is 377-fold higher than that for the sensitive parasite [44]. Parasites were screened overnight with either 2 or 4 µg/mL PZQ; the excess PZQ was washed out, and the schistosomes were cultured for 3 days. Those that survived after 3 days were considered resistant and placed in cell culture plates. Each screen contained 10 worms per well and was performed in three biological replicates (95% confidence intervals shown), treated with CIDD-0150303, and followed for 14 days. As CIDD-0150303 shares the same target as OXA, co-treatment with PZQ may reduce the risk for future CIDD-0150303 resistance. Because schistosomes are dioecious multicellular eukaryotic parasites, the adult worms only reproduce sexually within the human body, thereby producing eggs. Since drug resistance to PZQ and OXA are double-recessive traits [44,101], it is unlikely that an adult male and an adult female would separately develop resistance to both drugs and produce resistant progeny.

**Figure 13:**
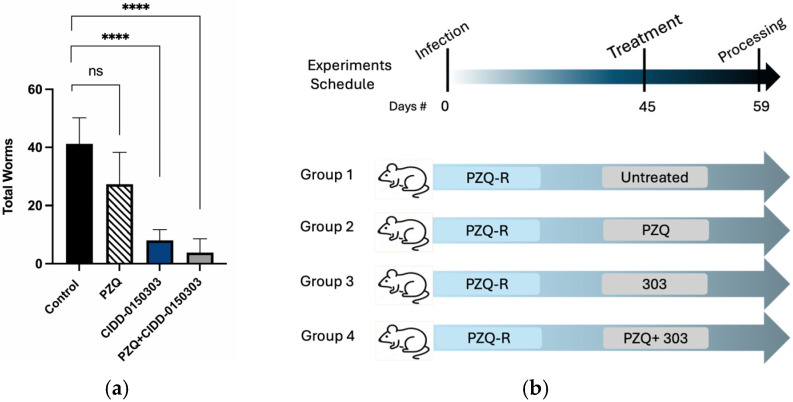
Combination therapy of PZQ and CIDD-0150303 against *Sm* PZQ-R parasite in vivo. (**a**) Combination therapy evaluation. (**b**) Experiment schedule. Five Balb/C mice per group were infected with 80 cercaria. Experimental groups were treated with 100 mg/kg of either OXA or PZQ orally for the single therapy and 100 mg/kg of both drugs for the combined therapy on 45 dpi orally. Mice were necropsied on 59 dpi. Worms were perfused from the portal and mesenteric veins with perfusion saline into Petri dishes and counted. Reductions and significance are represented relative to untreated control. PZQ + CIDD-0150303 reduced worm burden by 90.8% (*p* = 0.0001) (95% CI), and CIDD-0150303 alone reduced worm burden by 80.5% (*p* = 0.0001) (95% CI). Prism/unpaired *t*-test (*p* < 0.05). *p* < 0.0001 (****). ns, not significant [70].

Follow-up animal experiments were performed. A total of 20 mice, each infected with 80 cercariae of a PZQ-R strain, were treated in groups of 5 mice after 45 dpi with PZQ, CIDD-0150303, CIDD-0150303 + PZQ, or diluent. Fourteen days later, mice were sacrificed, and the worm burden was determined. Mice were treated with 100 mg/kg of each drug in 100 mL. In the CIDD-0150303 + PZQ group, the mice were treated with 200 mg/kg of total drugs in 100 mL. The drugs were mixed together, remaining soluble, and were given as one oral co-dose by gavage. The graph below (Figure 13) shows that the PZQ-R parasites are indeed resistant to PZQ treatment. The combination of PZQ and CIDD-0150303 reduced worm burden by 90.8% (*p* = 0.0001, 95% CI), and CIDD-0150303 alone reduced worm burden by 80.5% (*p* = 0.0001, 95% CI). Importantly, the level of killing for CIDD-0150303 was comparable to PZQ/CIDD-0150303, indicating that CIDD-0150303 is effective against both sensitive and resistant schistosome populations. A summary of in vivo worm burden reductions is provided in supplementary Table S3.

These findings support the potential of CIDD-0150303 to be used in combination with PZQ to enhance treatment efficacy and possibly reduce the likelihood of resistance emerging. The long-term goal is to achieve 100% killing of adult worms and to improve co-dosing efficacy against liver-stage parasites, which would represent a meaningful advance over PZQ monotherapy.

A key limitation of this study is the absence of formal safety and toxicity data for CIDD-0150303. While preliminary in vivo efficacy studies demonstrate that a single oral dose of 100 mg/kg can reduce worm burden by up to 80%, additional animal studies are required to establish the therapeutic index and tolerability profile. Future work will focus on expanding toxicity studies, optimizing compound properties through medicinal chemistry, and performing detailed PK/PD analyses to support IND-enabling studies. These efforts are expected to determine whether CIDD-0150303 can achieve the ultimate goal of complete clearance of worms and eggs in vivo, thereby reducing reinfection and blocking transmission.

## 4. Conclusions

Taking an iterative approach to identify derivatives of OXA has resulted in significant advances over current therapies. CIDD-0150303’s cidal efficacy against the two main human schistosome species is a major improvement over OXA. Similarly, efficacy against early and advanced infection is a major advancement over PZQ. Importantly, producing a drug with a different mode of action that can be co-dosed with PZQ to improve efficacy and mitigate the development of drug resistance is significant. The drug can be further improved by increasing solubility, permeability, and metabolic liabilities, which will be key to developing a highly effective compound. This will also allow the dose to be lowered, which should improve off-target tolerability related to dizziness and sedation, which currently limits OXA dosing levels.

## 5. Patents

McHardy, S.F., LoVerde, P.T., Taylor, A.B., Tarpley, R. & Anderson, T.J. Composition and methods for treating Schistosoma infection. Date: 29 April 2019. Number: WO2020223370A1. Status: Filed. URL: https://patents.google.com/patent/WO2020223370A1/ (accessed on 15 September 2025).

## Data Availability

The data supporting the findings of this study are available in previously published articles: [70,86,89,92,93]. No new datasets were generated or analyzed in this study.

## Supplementary Materials

The following supporting information can be downloaded at https://www.mdpi.com/article/10.3390/pharmaceutics17101268/s1, Table S1: Statistical analysis of worm burden in mice treated with CIDD-0150303 (Figure 9a,b); Table S2: Statistical analysis of worm burden in mice treated with CIDD-0150303 at different infection stages (Figure 10); Table S3: Statistical analysis of worm burden in treated vs. control animals (13); Table S4: In vitro and in vivo efficacy of CIDD-0150303 against Schistosoma life stages and species.

## Abbreviations

The following abbreviations are used in this manuscript:

PZQ	Praziquantel
OXA	Oxamniquine
*Sm*	*Schistosoma mansoni*
*Sh*	*S. haematobium*
DALYs	Disability-adjusted life year
MDA	Mass drug administration
TRPMPZQ	Transient receptor potential channel
HYC	Hycanthone
*Sm*SULT	*S. mansoni* sulfotransferase
PAPS	3′-phosphoadenosine 5′-phosphosulfate
SULT	Sulfotransferase
*Sh*SULT	*S. haematobium* sulfotransferase
qPCR	Quantitative polymerase chain reaction
PZQ-R	PZQ-resistant
