# Supplementary material for: Development of a Novel Compound Effective Against Juvenile, Adult, and Drug-Resistant Schistosoma Species
Source: Pharmaceutics. 2025 Sep 27;17(10):1268. doi: 10.3390/pharmaceutics17101268 (PMC12567056; doi:10.3390/pharmaceutics17101268)
Supplement: Supplementary file 1 [file pharmaceutics-17-01268-s001.zip › pharmaceutics-3834125-supplementary.pdf]

pharmaceutics

Development of a Novel Compound Effective Against Juvenile, Adult, and Drug-Resistant *Schistosoma* Species

Sevan N. Alwan [1,*], Alexander B. Taylor [1,2], Stanton F. McHardy [3], Michael D. Cameron [4] and Philip T. LoVerde [1]

Supplementary Tables:

Supplementary Table S1. Statistical analysis of worm burden in mice treated with CIDD-0150303 (Figure 9A–B).

Comparison	Species	Mean ± SEM (Control)	Mean ± SEM (Treatment)	t, df	P value	95% CI of difference	R^2 (η^2)	Significance
OXA vs Control	S. mansoni	66.25 ± SEM	4.20 ± SEM	t=6.934, df=7	0.0002	-83.21 to -40.89	0.8729	***
CIDD-0150303 vs Control	S. mansoni	66.25 ± SEM	12.00 ± SEM	t=5.379, df=6	0.0017	-78.93 to -29.57	0.8282	**
CIDD-0150303 vs Control	S. haematobium	81.00 ± SEM	32.33 ± SEM	t=3.182, df=5	0.0245	-87.98 to -9.355	0.6695	*

Notes:

- Unpaired two-tailed t test was used for all comparisons.
- Variance between groups was not significantly different (F test, $p > 0.05$).
- Significance codes: **** $p < 0.0001$; *** $p < 0.001$; ** $p < 0.01$; * $p < 0.05$; ns, not significant.
- n = 3–5 mice per group as indicated.

Supplementary Table S2. Statistical analysis of worm burden in mice treated with CIDD-0150303 at different infection stages (Figure 10).

Comparison	Mean ± SEM (Untreated)	Mean ± SEM (Treatment)	t, df	P value	95% CI of difference	R^2 (η^2)	Significance
20 dpi vs Untreated	56.67 ± SEM	38.75 ± SEM	t=1.810, df=5	0.1300	-43.36 to 7.522	0.3960	ns
25 dpi vs Untreated	56.67 ± SEM	20.50 ± SEM	t=19.44, df=5	<0.0001	-40.95 to -31.38	0.9869	****
28 dpi vs Untreated	56.67 ± SEM	29.00 ± SEM	t=20.75, df=4	<0.0001	-31.37 to -23.96	0.9908	****
32 dpi vs Untreated	56.67 ± SEM	26.00 ± SEM	t=16.35, df=3	0.0005	-36.64 to -24.70	0.9889	***

 https://doi.org/10.3390/pharmaceutics17101268

Notes:
- Unpaired two-tailed t test was used for all comparisons.
- Variance between groups was significantly different only for 20 dpi vs untreated (F test, p = 0.0167).
- Significance codes: **** $p < 0.0001$; *** $p < 0.001$; ns, not significant.
- n = 2–4 mice per group as indicated.

Supplementary Table S3. Statistical analysis of worm burden in treated vs. control animals (13).

Comparison	Mean ± SEM (Control)	Mean ± SEM (Treatment)	t, df	P value	95% CI of difference	R^2 (η^2)	Significance
PZQ vs Control	41.20 ± SEM	27.40 ± SEM	t=2.187, df=8	0.0602	-28.35 to 0.7479	0.3743	ns
CIDD-0150303 vs Control	41.20 ± SEM	8.00 ± SEM	t=7.669, df=8	<0.0001	-43.18 to -23.22	0.8803	****
PZQ + CIDD-0150303 vs Control	41.20 ± SEM	3.80 ± SEM	t=8.244, df=8	<0.0001	-47.86 to -26.94	0.8947	****

Notes:
- Unpaired two-tailed t test was used for all comparisons.
- Variance between groups was not significantly different (F test, $p > 0.05$ for all).
- Significance codes: **** $p < 0.0001$; ns, not significant.
- n = 5 mice per group.

Supplementary Table S4. *In vitro* and *in vivo* efficacy of CIDD-0150303 against Schistosoma life stages and species.

Compound	Species	Life Stage Tested	*In vitro* killing at (71.5 μM)	*In vivo* % Reduction (mean ± SEM)
CIDD-0150303	S. mansoni	Adult	100%	81.9%
		Juvenile	100%	71.2%
	S. haematobium	Adult	100%	64.7%
		Juvenile	100%	NA
